# The Neuroprotective Effect of Thiopental on the Postoperative Neurological Complications in Patients Undergoing Surgical Clipping of Unruptured Intracranial Aneurysm: A Retrospective Analysis

**DOI:** 10.3390/jcm10061197

**Published:** 2021-03-12

**Authors:** Byung-Gun Kim, Young-Tae Jeon, Jiwon Han, Yu Kyung Bae, Si Un Lee, Jung-Hee Ryu, Chang-Hoon Koo

**Affiliations:** 1 Department of Anesthesiology and Pain Medicine, Inha University School of Medicine, Inha University Hospital, Incheon 22332, Korea; wangunlone@gmail.com; 2 Department of Anesthesiology and Pain Medicine, Seoul National University College of Medicine, Seoul 03080, Korea; ytjeon@snubh.org (Y.-T.J.); jinaryu74@gmail.com (J.-H.R.); 3 Department of Anesthesiology and Pain Medicine, Seoul National University Bundang Hospital, Seongnam 13620, Korea; hanjiwon@snubh.org (J.H.); vansuri27@gmail.com (Y.K.B.); 4 Department of Neurosurgery, Seoul National University Bundang Hospital, Seongnam 13620, Korea; bigmidget@hanmail.net

**Keywords:** intracranial aneurysm, neurosurgery, postoperative complications, thiopental

## Abstract

Although thiopental improved neurological outcomes in several animal studies, there are still insufficient clinical data examining the efficacy of thiopental for patients undergoing surgical clipping of unruptured intracranial aneurysm (UIA). This study validated the effect of thiopental and investigated risk factors associated with postoperative neurological complications in patients undergoing surgical clipping of UIA. In total, 491 patients who underwent aneurysm clipping were included in this retrospective cohort study. Data regarding demographics, aneurysm characteristics, and use of thiopental were collected from electronic medical records. Propensity score matching and logistic regression analysis were used. After propensity score matching, the thiopental group showed a lower incidence of the postoperative neurological complications than non-thiopental group (5.5% vs. 17.1%, *p* = 0.001). In multivariate analysis, thiopental reduced the risk of postoperative neurological complications (odds ratio (OR) 0.26, 95% confidence interval (CI) 0.13 to 0.51, *p* < 0.001) while aneurysm size ≥ 10 mm (OR 4.48, 95% CI 1.69 to 11.87, *p* = 0.003), and hyperlipidemia (OR 2.24, 95% CI 1.16 to 4.32, *p* = 0.02) increased the risk of postoperative neurological complications. This study showed that thiopental was associated with the lower risk of neurological complications after clipping of UIA.

## 1. Introduction

Unruptured intracranial aneurysm (UIA) has a reported incidence of up to 3% in the general population [1]. Surgical clipping is considered suitable for the treatment of UIA. During surgical clipping of UIA, temporary clipping of parent artery is commonly performed to soften the UIA, thereby reducing intraoperative rupture and facilitating approach to vessels [2,3]. However, temporary clipping is associated with cerebral ischemia and postoperative neurological complications [4,5,6]. Cerebral infarction occurs in approximately 11% of patients who have undergone surgical clipping of UIA [7]. Some measures, such as hypothermia, neurophysiologic monitoring, or pharmacological interventions, have been suggested to prevent neurological impairment after UIA clipping [8,9,10].

Thiopental, as a pharmacological treatment, has been known to have neuroprotective effect of brain over the years [11,12]. Thiopental induces burst suppression, which decreases cerebral metabolism and redistributes cerebral blood flow [13]. However, this neuroprotective effect of thiopental was addressed only in animal models [11]. There has been little discussion on the neuroprotective effect of thiopental in humans.

Since postoperative neurological complications are associated with mortality, morbidity, and prolonged length of hospital stay, several studies have established risk factors for postoperative neurological complications in patients undergoing surgical clipping of UIA [14,15]. However, those studies rarely considered the effect of thiopental on the postoperative neurological complications. Therefore, this study was performed to establish the clinical efficacy of thiopental during surgical clipping of UIA and to investigate the risk factors associated with postoperative neurological complications after clipping of UIA.

## 2. Materials and Methods

### 2.1. Study Design

This study was approved by the Institutional Review Board (IRB) of Seoul National University Bundang Hospital, Seongnam, Korea (IRB approval number: B-1710/427-113). The requirement for informed consent was waived due to the retrospective nature of the study design. Patients who had been diagnosed with UIA and had undergone surgical clipping at our institution between May 2003 and August 2016 were included in this study. The exclusion criteria were as follows: (1) Concomitant brain tumor that would affect postoperative neurological complications, and (2) UIA associated with arteriovenous malformation or arteriovenous fistula.

### 2.2. Data Collection

The following data were collected from electronic medical records: Sex, age, height, weight, smoking history, alcohol use, concomitant diseases, size of aneurysm, number of aneurysms, locations of aneurysms, anesthesia time, operation time, intraoperative hemodynamics, intravenous thiopental administration, and laboratory data. Aneurysm size was defined as the larger of width or height according to the previous study [16]. Aneurysms were categorized according to size (i.e., <5 mm, 5–10, or ≥10 mm), number (i.e., 1 or >1), and location (i.e., middle cerebral artery, internal carotid artery, anterior communicating artery, other anterior circulation, posterior circulation, or >2 sites). A neurosurgeon who was not involved in the surgeries determined the presence of postoperative neurological complications, including transient ischemic attack or acute infarction.

### 2.3. Surgery and Anesthesia

Experienced neurosurgeons performed surgical clipping, using standard craniotomy procedures, in patients who had UIA with bleb, those who had uncoilable UIA due to location or shape, or those who refused to receive endovascular coiling. Temporary clips were routinely placed to soften the aneurysm and to reduce intraoperative rupture. A bolus of 200 mg of thiopental was administered immediately before application of the temporary clip at the attending surgeon’s discretion. Anesthesia was induced and maintained by total intravenous anesthesia using propofol and remifentanil.

### 2.4. Statistical Analysis

Demographic variables are expressed as means ± standard deviations (continuous variables) or number with percentage (categorical variables). To minimize the effect of confounding variables, propensity score (PS) matching was performed using the nearest neighbor method at a 1:1 ratio, without replacement and a caliper of 0.2. PS was calculated by logistic regression analysis including all preoperative variables, intraoperative variables, and characteristics of UIA. Univariate logistic regression analysis was performed to identify risk factors for postoperative neurological complications. Subsequently, variables with *p* < 0.2 in univariate analysis were included in multivariate analysis using forward selection. In all analyses, *p* < 0.05 was considered to indicate statistical significance. Statistical analyses were performed using SPSS Statistics, version 24 (IBM Corp., Armonk, NY, USA).

## 3. Results

In total, 491 patients who had undergone surgical clipping of UIA were enrolled in this study. Fifty-one out of 491 patients (10.4%) experienced neurological complications after clipping surgery of UIA. Among the total of 491 patients, 263 patients were administered thiopental (TPT group) during surgical clipping while 228 patients did not receive thiopental during surgery (Non-TPT group). After PS matching, 181 patients were allocated to each group. Table 1 represents the results of comparative analysis of the two groups between and after PS matching. After matching, standardized mean difference, less than 0.2, indicated well balance and significant differences between two groups were not observed in all variables (*p* > 0.05).

As shown in Table 2, postoperative neurological complications were significantly lower in the TPT group than the non-TPT group. The significant difference was found not only in the entire cohort analysis (odds ratio (OR) 0.26, 95% confidence interval (CI) 0.13 to 0.50, *p* < 0.001), but also in the PS-matched cohort analysis (OR 0.28, 95% CI 0.13 to 0.60, *p* = 0.001).

The results of univariate and multivariate logistic regression analysis are shown in Table 3 and Table 4. The result of multivariate analysis showed that intraoperative thiopental administration significantly reduced the postoperative neurological complications (OR 0.26, 95% CI 0.13 to 0.51, *p* < 0.001). The multivariate model revealed the risk factors of postoperative neurological complications in patients that underwent surgical clipping of UIA aneurysm size ≥ 10 mm (OR 4.48, 95% CI 1.69 to 11.87, *p* = 0.003) and hyperlipidemia (OR 2.24, 95% CI 1.16 to 4.32, *p* = 0.02). The multivariate model showed an adequate goodness-of-fit, assessed by Hosmer and Lemeshow test (*p* = 0.43).

## 4. Discussion

This retrospective analysis showed that intraoperative thiopental administration significantly reduced the postoperative neurological complications in patients undergoing surgical clipping of UIA. This neuroprotective effect of thiopental was also observed in the multivariate model of entire cohort. This study also showed that aneurysm size and hyperlipidemia were independently associated with postoperative neurological complications in patients who underwent UIA clipping.

This study proved the neuroprotective effect of thiopental in patients undergoing surgical clipping of UIA. Thiopental administration has been described as an effective method to prevent postoperative neurological deficits in several animal studies [17,18,19,20,21]. There are several proposed mechanisms for cerebral protection of thiopental. First, thiopental improves cerebral oxygenation. Thiopental modulates NMDA receptors, depressing the inward current [22]. Subsequently, it reduces the cerebral metabolic rate of oxygen, thereby decreasing oxygen consumption in ischemic area [21]. Second, thiopental causes “inverse steal” phenomenon, a redistribution of cerebral blood flow from hyper-perfused region to hypo-perfused region [23]. Third, thiopental have an anti-oxidative effect through inhibiting hydroxyl radical generation and lipid peroxidation [24]. However, the clinical efficacy of thiopental for human still remains unclear. Several studies have shown that the duration of burst suppression is insufficient to protect against cerebral ischemia [25,26]. In these previous studies, inhalation anesthetics were used to maintain anesthesia. Interestingly, the most remarkable point of the present study is that intravenous anesthetics were administered throughout the operation. Total intravenous anesthesia has been reported to provide a longer duration and higher degree of burst suppression, compared to inhalational anesthetics [27]. This factor appears to be responsible for favorable effect of thiopental in the present study. To the best of our knowledge, this study is the first study to support neuroprotective effect of thiopental in patients who had UIA clipping surgery under total intravenous anesthesia.

Aneurysm size ≥ 10 mm was significantly associated with poor neurological outcome in the present study. In previous studies, large aneurysm size was associated with surgery-related mortality and morbidity [28,29]. Similar to our findings, large aneurysm size has been shown to predict postoperative neurological complications in patients undergoing not only surgical clipping [28,29,30], but also endovascular coiling for UIA [31]. Aneurysm size ≥ 10 mm was associated with an approximately three-fold increase in the rate of postoperative neurological complications. These findings suggest that large aneurysms may increase thromboembolic risk, thereby causing neurological deficits [32]. Another possible explanation is that large aneurysms may provide intracranial mass and unexpected tissue transformation [33]. Large UIA is challenging for neurosurgeons due to thick and atheromatous wall, intramural thrombus, calcification, and incorporated perforating arteries [2]. Temporary clipping facilitates surgical procedures, that is, safe dissection of UIA and preservation of other vessels [34]. During the temporary clipping, thiopental administration promotes collateral circulation and reduces the risk of postoperative neurological complications [34]. A previous report insisted that temporary clipping with barbiturate administration may be a safe technique for large UIA [34].

As reported by previous studies [35,36], hyperlipidemia was an independent risk factor for postoperative neurological complications. This may be attributed to vascular fragility and atherosclerosis, inducing reduction in cerebral blood flow and cerebral perfusion [36,37,38].

Although previous studies found that age is an important risk factor associated with surgical outcomes, the current study does not support previous result in this area. The risk for surgery substantially increases for patients about 50 years and older [7]. Therefore, it is recommended to consider the patient’s age on the decision of treatment. Contrarily, age seems to be insignificant factor for postoperative neurological complications in this study. This discordant result may be explained by a great proportion of old patients (≥50) in this study. A total of 83.1% of included patients were ≥ 50, thus more patients who are <50 may be needed to show the effect of age on postoperative neurological complications in this study. On the other hand, apart from this disagreement, our finding appears to be well substantiated by several studies reporting insignificant association between age and surgical outcomes [28,39,40].

This study had some limitations. First, neurological complications were evaluated only during the hospital stay; therefore, further studies with long-term follow-up are needed to confirm the findings. Second, temporary clip may be a risk factor for postoperative neurological complications, thus data such as duration and number of temporary clips had been tried to be collected. However, it was impossible to reveal the effect of temporary clip because information had not been recorded in detail. Further studies regarding temporary clip may be needed.

## 5. Conclusions

In conclusion, this retrospective study showed that intraoperative thiopental administration was associated with a reduction in the postoperative neurological complications in patients undergoing surgical clipping of UIA. The finding of the present study provides a considerable clinical implication that use of thiopental may be a reasonable strategy to prevent cerebral ischemia or infarction during UIA surgery. Further prospective randomized trials are encouraged to validate the results of the present study.

## Figures and Tables

**Table 1:** Comparisons of demographic variables before and after propensity score matching.

Variables	Entire Cohort (*n* = 491)			PS-Matched Cohort (*n* = 362)		
	TPT *n* = 263	Non-TPT *n* = 228	SMD	TPT *n* = 181	Non-TPT *n* = 181	SMD
Preoperative variables						
Sex: Female	185 (70.3%)	148 (64.9%)	0.116	132 (72.9%)	118 (65.2%)	0.168
Age (Year)	59.9 ± 10.4	59.0 ± 9.9	0.091	59.4 ± 9.7	59.2 ± 9.8	0.023
Height (cm)	159.2 ± 9.7	159.5 ± 8.5	0.032	158.6 ± 10.3	159.3 ± 8.4	0.067
Weight (kg)	61.8 ± 10.6	62.4 ± 10.7	0.056	61.7 ± 10.5	62.0 ± 11.0	0.031
Smoker	31 (11.8%)	43 (18.9%)	0.197	28 (15.5%)	30 (16.6%)	0.030
Alcohol	30 (11.4%)	40 (17.5%)	0.175	25 (13.8%)	31 (17.1%)	0.092
Hypertension	139 (52.9%)	126 (55.3%)	0.048	102 (56.4%)	101 (55.8%)	0.011
Diabetes Mellitus	28 (10.6%)	24 (10.5%)	0.004	20 (11.0%)	20 (11.0%)	<0.001
Coronary Heart Disease	8 (3.0%)	12 (5.3%)	0.112	7 (3.9%)	8 (4.4%)	0.028
Thyroid disease	8 (3.0%)	16 (7.0%)	0.183	8 (4.4%)	10 (5.5%)	0.051
Hyperlipidemia	46 (17.5%)	48 (21.1%)	0.090	37 (20.4%)	36 (19.9%)	0.014
Characteristics of UIA						
Aneurysm size (mm)			0.176			0.047
<5	146 (55.5%)	143 (62.7%)		108 (59.7%)	112 (61.9%)	
5≤ <10	105 (39.9%)	72 (31.6%)		65 (35.9%)	61 (33.7%)	
≥10	12 (4.6%)	13 (5.7%)		8 (4.4%)	8 (4.4%)	
Multiplicity			0.315			0.041
1	186 (70.7%)	191 (83.8%)		143 (79.0%)	146 (80.7%)	
>1	77 (29.3%)	37 (16.2%)		38 (21.0%)	35 (19.3%)	
Location			0.563			0.179
MCA	181 (68.8%)	108 (47.4%)		108 (59.7%)	99 (54.7%)	
ICA	5 (1.9%)	4 (1.8%)		4 (2.2%)	4 (2.2%)	
Acom	20 (7.6%)	37 (16.2%)		20 (11.0%)	26 (14.4%)	
Other anterior circulation	25 (9.5%)	51 (22.4%)		23 (12.7%)	31 (17.1%)	
Posterior circulation	0 (0.0%)	5 (2.2%)		26 (14.4%)	21 (11.6%)	
More than 2 sites	32 (12.2%)	23 (10.1%)		108 (59.7%)	99 (54.7%)	
Intraoperative variables						
Anesthesia time (min)	330.3 ± 83.7	294.3 ± 76.3	0.449	302.2 ± 52.1	302.1 ± 79.4	0.001
Operation time (min)	265.6 ± 80.3	230.3 ± 72.7	0.461	238.7 ± 49.4	237.9 ± 75.7	0.012
Highest SBP (mmHg)	147.7 ± 16.4	145.9 ± 22.7	0.092	147.9 ± 17.8	146.8 ± 23.2	0.054
Lowest SBP (mmHg)	87.5 ± 9.7	86.1 ± 9.1	0.155	86.2 ± 9.7	86.2 ± 9.1	0.001
Estimated blood loss (mL)	577.6 ± 460.2	469.5 ± 334.2	0.269	471.3 ± 252.1	475.6 ± 318.8	0.015
Laboratory variables						
Preoperative Hb (g/dL)	13.6 ± 1.4	13.7 ± 1.4	0.035	13.6 ± 1.3	13.7 ± 1.3	0.041
Postoperative Hb (g/dL)	11.4 ± 1.5	11.5 ± 1.3	0.066	11.4 ± 1.5	11.4 ± 1.3	0.006
Preoperative WBC (10^3^/µL)	6.7 ± 2.5	6.4 ± 1.9	0.131	6.7 ± 2.3	6.5 ± 2.0	0.131
Postoperative WBC (10^3^/µL)	8.7 ± 3.8	7.7 ± 3.2	0.287	8.3 ± 3.6	7.7 ± 3.1	0.150

Abbreviations: SMD, standardized mean difference; UIA, unruptured intracranial aneurysm; MCA, middle cerebral artery; ICA, internal carotid artery; Acom, anterior communicating artery; SBP, systolic blood pressure; Hb, hemoglobin; WBC, white blood cell. Data are expressed as mean ± SD or number (%).

**Table 2:** Postoperative neurological complications after surgical clipping of UIA before and after propensity score matching.

Variables	Event Rate	OR (95% CI)	*p* Value
Entire cohort (before PSM)			<0.001
Non-TPT group	38 of 228 (16.7%)	1	
TPT group	13 of 263 (4.9%)	0.26 (0.13 to 0.50)	
PS-matched cohort (After PSM)			
Non-TPT group	31 of 181 (17.7%)	1	0.001
TPT group	10 of 181 (5.5%)	0.28 (0.13 to 0.60)	

Abbreviations: PSM, propensity score matching; OR, odds ratio; TPT, thiopental.

**Table 3:** Univariate logistic regression analysis of risk factors for postoperative neurological complications.

Variables	Odds Ratio (95% CI)	*p* Value
Demographic data		
Sex	0.96 (0.51 to 1.79)	0.90
Age	1.02 (0.99 to 1.05)	0.25
Height	1.00 (0.97 to 1.03)	0.98
Weight	1.01 (0.98 to 1.04)	0.57
Hypertension	1.25 (0.69 to 2.24)	0.46
Diabetes Mellitus	1.98 (0.90 to 4.34)	0.09
Coronary heart disease	0.96 (0.22 to 4.25)	0.95
Thyroid disease	1.25 (0.36 to 4.34)	0.73
Hyperlipidemia	2.36 (1.25 to 4.44)	0.008 *
Characteristics of Aneurysm		
Size		
<5	1	
5≤ <10	0.86 (0.45 to 1.66)	0.65
≥10	4.39 (1.74 to 11.08)	0.002 *
Multiplicity		
1	1	
> 1	1.14 (0.58 to 2.22)	0.71
Location		
MCA	1	
Acom	0.97 (0.36 to 2.65)	0.96
Other anterior circulation	1.87 (0.90 to 3.90)	0.10
ICA/Posterior circulation	-	-
More than 2 sites	1.72 (0.74 to 4.03)	0.21
Intraoperative variables		
Anesthesia time (min)	1.00	-
Operation time (min)	1.00	-
Highest SBP (mmHg)	1.00 (0.99 to 1.02)	0.78
Lowest SBP (mmHg)	1.00 (0.97 to 1.03)	0.88
Use of thiopental	0.26 (0.14 to 0.50)	<0.001 *
Estimated blood loss (mL)	1.00	-
Laboratory variables		
Preoperative Hb (g/dL)	0.96 (0.78 to 1.18)	0.70
Postoperative Hb (g/dL)	1.07 (0.87 to 1.32)	0.52
Preoperative WBC (10^3^/µL)	0.87 (0.74 to 1.02)	0.09
Postoperative WBC (10^3^/µL)	0.97 (0.89 to 1.05)	0.43

CI, confidence intervals; MCA, middle cerebral artery; Acom, anterior communicating artery; ICA, internal carotid artery; SBP, systolic blood pressure. * *p* < 0.05.

**Table 4:** Multivariate logistic regression analysis of risk factors for postoperative neurological complications.

Variables	Odds Ratio (95% CI)	*p* Value
Use of thiopental	0.26 (0.13 to 0.51)	<0.001
Size of aneurysm ≥ 10 mm	4.48 (1.69 to 11.87)	0.003
Hyperlipidemia	2.24 (1.16 to 4.32)	0.02

CI, Confidence intervals; RBC, Red blood cell.

## Data Availability

The datasets generated and analyzed during the current study are available on request to the corresponding author on reasonable request.
